# Youth and Parent Experiences in a Multidisciplinary Gender Clinic

**DOI:** 10.1089/trgh.2018.0046

**Published:** 2019-04-02

**Authors:** David J. Inwards-Breland, Sara DiVall, Parisa Salehi, Julia M. Crouch, Morgan Negaard, Amanda Lu, Alena Kantor, Katie Albertson, Kym R. Ahrens

**Affiliations:** ^1^ Center for Child Health, Behavior and Development, Seattle Children's Research Institute, Seattle, Washington.; ^2^ Division of Adolescent Medicine, Seattle Children's Hospital, Seattle, Washington.; ^3^ Division of Endocrinology, Seattle Children's Hospital, Seattle, Washington.; ^4^ Department of Pediatrics and Adolescent Medicine, School of Medicine, University of Washington, Seattle, Washington.; ^5^ The Warren Alpert Medical School, Brown University, Providence, Rhode Island.

**Keywords:** gender clinic, gender nonconforming, multidisciplinary, satisfaction, transgender, youth

## Abstract

**Purpose:** To assess youth and parent/caregiver satisfaction with care at a pediatric multidisciplinary gender clinic.

**Methods:** Transgender/gender nonconforming youth (*n*=33) and their parent/caregiver (*n*=29) completed self-report questionnaires and individual interviews (*n*=20) about experiences and satisfaction with care.

**Results:** Quantitatively, participants reported being extremely satisfied with care experiences (parents 97%; youth 94%). Qualitatively, main themes included (1) affirmation due to use of preferred name/pronouns, (2) access barriers due to scheduling and readiness assessments, and (3) positive interactions with Care Navigator.

**Conclusion:** Youth and parents/caregivers are highly satisfied with multidisciplinary, coordinated health care for transgender/gender nonconforming youth; however, some challenges remain.

## Background

Transgender/gender nonconforming (TGNC) youth face barriers to gender-affirming medical, surgical, and mental health care, including a dearth of providers trained or knowledgeable in delivering gender-affirming care for youth, uncoordinated care resulting in access delays to crucial gender-affirming medical interventions (e.g., pubertal blockers, cross-sex hormones), and inconsistent use of patients' chosen name and pronoun.^1^ Echoing that final barrier, a recent study by Russell et al. suggests that among transgender youth, ability to use chosen name across a variety of settings may reduce potential mental health risks (e.g., depression, suicidal ideation and behavior).^2^ Recently, several U.S. pediatric institutions have opened multidisciplinary gender clinics.^3,4^ Despite the potential for these clinics to improve the lives of TGNC youth and families, no studies have explored the satisfaction of their patients and families. The multidisciplinary Seattle Children's Gender Clinic (SCGC) opened in 2016. SCGC is composed of a Care Navigator (social worker who helps families navigate complex practical and legal aspects of transitioning), adolescent medicine and endocrine specialists, mental health providers, nurses, and medical assistants. The clinic structure was designed to address concerns noted in our earlier study and described previously.^1^ All families complete a phone intake with the Care Navigator and, if interested, are offered a medical intake appointment. The medical intake appointment includes assessment of medical comorbidities, pubertal staging, and patient/family education.

SCGC works with community mental health therapists who complete a readiness questionnaire for new patients, assessing for gender dysphoria, mental health comorbidities, possible autism spectrum disorder, resilience, potential barriers to ongoing gender-affirming care, parental support, and recommended treatment plan. The SCGC mental health provider assesses the same general considerations for those patients who prefer to have their readiness assessment done at our clinic rather than by a community therapist. When patients are in need of ongoing mental health support, the SCGC Care Navigator will help families' access mental health providers in the community. We currently recommend but do not require ongoing therapy for minor patients for at least 1 year after beginning the treatment. If patients are young adults (≥18 years old) at the time of intake, we provide gender-affirming care using an informed consent model. Current guidelines do not require an assessment or ongoing therapy by a mental health provider.^5^ The Care Navigator can also provide resources for ongoing mental health therapy for parents/caregivers when needed.

This study evaluated patient and family satisfaction during the first year of SCGC.

## Methods

### Recruitment

Patients and parents/caregivers were recruited from the SCGC during clinic visits between November 2016 and March 2017. Patients were eligible to participate in the study if they were receiving care at SCGC and were between ages 8 and 22. In an effort to obtain the experiences and opinions of both patients and parents/caregivers regarding access to and satisfaction with multidisciplinary gender care, patients under 18 needed a parent to participate along with them. The study was approved by the Seattle Children's Institutional Review Board and written consent/assent was obtained.

### Data collection

Data for this study were collected using a mixed-methods approach, including a web-based survey and qualitative interviews. Forty-nine TGNC youth and 42 parents/caregivers were approached during SCGC visits by research staff, presented with information about the study, and offered the option to participate. Of those approached, 33 TGNC youth and 29 parents/caregivers (*n*=62) completed the web-based survey (Table 1) through REDCap, a secure web application for building/managing online surveys.

**Table 1.** Data Collection Response

Online survey	*n*
Overall sample	
Approached	91
Consented/sent survey link	72
Completed survey	62
Declined or otherwise not complete survey	19
Youth	
Approached	49
Consented/sent survey link	38
Completed survey	33
Declined or otherwise not complete survey	11
Caregiver	
Approached	42
Consented/sent survey link	33
Completed survey	29
Declined or otherwise not complete survey	8
Phone interview	
Youth	
Interested in being interviewed^a^	27
Skipped the question	6
Completed interview	10
Caregiver	
Interested in being interviewed^a^	24
Skipped the question	5
Completed interview	10

^a^ Includes response option “Maybe.”

The research team developed and adapted survey questions to assess experiences of TGNC youth and their parents/caregivers regarding accessing gender-affirming care, satisfaction with specific aspects of care (e.g., Care Navigation, mental health assessments and therapy referrals, puberty blockers, cross-sex hormones, and surgery referrals), and overall impressions of SCGC. The survey's demographics section consisted of a series of standard questions assessing race/ethnicity, sex assigned at birth, gender identity, age, insurance status/type, and language spoken at home, among other items. Experiences at SCGC and with gender-related care were assessed using a mix of 5-point Likert scales, multiple choice response options, and open-ended questions. Participants aged 8–12 were not queried about services they received at SCGC; the research team believed it was more appropriate to ask parents/caregivers of these participants about the services their child received. Once informed consent/assent was obtained, study staff obtained the preferred e-mail address of the youth and/or parents/caregivers. A link to the secure survey was sent through e-mail ∼1–2 months after each participant's enrollment time point (January through April 2017). Participants were able to complete the survey at their convenience and in the location of their choice once the survey link was received.

Qualitative interviews were conducted with a subset of 20 survey respondents (10 youth and 10 parents/caregivers). At the end of the survey, respondents were queried using a three-item response question (Yes, No, and Maybe) regarding interest in an optional phone or in-person interview. Among those who answered “Yes,” we used purposive sampling to ensure that the subset of participants interviewed was diverse in terms of the satisfaction scores they reported on their survey as well as their age, gender identity, and race/ethnicity. The interviews were conducted and digitally recorded by two cisgender female members of our research team, one of whom identifies as Asian American and the other as white. The interviewers were not clinical providers. The interview guide was designed to present participants with open-ended questions to explore aspects of the care experience and allow participants to provide additional contextual information. The guide consisted of ∼10 main questions (Table 2) and each interview lasted about 30 min. Participants received $20 for the survey and $30 for the interview.

**Table 2.** Interview Questions

Construct	Question and sample prompts
Gender exploration	Tell me about your (child's) gender journey?
Learning about gender care SCGC	Tell me about how you first got connected to the Seattle Children's Gender Clinic.
	What was your first interaction with the clinic staff like?
Overall experiences at SCGC	Overall, how satisfied are you with the care (you have/your child has) received at the gender clinic?
	In what ways have you satisfied with the care? Tell me more about that.
	What aspects of your care have been the most challenging for you?
	What about your experience so far at Seattle Children's Gender Clinic do you wish had been different?
	What barriers to care, if any, did you run into?
	What do you wish the gender clinic would do differently in the future to help provide the best possible care for future patients?
Experiences with specific SCGC services	
Care navigation	Tell me about (your/your child's) interactions with the gender clinic's Care Navigator, [NAME].
	How helpful (or not helpful) were they?
	Can you describe one way that they helped you navigate (your/your child's) care?
	Can you think of something you wish the Care Navigator had been more helpful with or done differently?
Readiness and mental health	If (you/your child) had a readiness evaluation with a mental health provider (also known as a gender dysphoria evaluation), can you tell me about that experience?
	Who did the evaluation with you? (At Children's, or with a someone outside Children's?)
	Would you say it was a positive or negative experience? Can you tell me about why you feel that way?
	If (you have/your child has) have received mental health therapy, or are interested in having ongoing therapy, can you tell me about that?
Puberty blockers and cross-sex hormone therapies	If (you have/your child has) received puberty blockers, or are interested in receiving puberty blockers, can you tell me about that?
	What could the gender clinic do to improve patients' experience in the future?
	If (you have/your child has) have received cross-hormones (testosterone or estrogen), or are interested in receiving cross-hormones, can you tell me about that?
	What could the gender clinic do to improve patients' experience in the future?
Gender-affirming surgery referrals	If (you have/your child has) have received gender-affirming surgery (outside of Seattle Children's), or are interested in getting surgery, can you tell me about that?

SCGC, Seattle Children's Gender Clinic.

### Analysis

We generated descriptive statistics from the survey data to capture basic demographics of our sample. Interviews were transcribed using a professional transcription company and reviewed for accuracy. An *a priori* framework was used to guide thematic analysis of interview data. Core domains included overall experiences at SCGC and satisfaction with specific gender-affirming care services: care navigation, mental health, puberty blockers, hormone replacement therapy, and gender-affirming surgery referrals. We used thematic analysis techniques to analyze the qualitative interview data.^6^ Two coders independently read and reread each interview transcript to develop and merge initial codes and create a codebook, and the study team members met weekly to discuss codes and themes and discuss/resolve any disagreements. Using Dedoose.com, we applied thematic codes to relevant transcript quotations and analyzed code queries to assess the significance of each theme.

## Results

The overall survey response rate among study participants was 86%. Mean age of youth was 16 and 55.6% identified as transgender male, 38.9% as transgender female, and 5.6% as nonbinary. Half identified as white or Caucasian (51.4%) and 28.6% as more than one race. The participant population demographics varied from that of the general Adolescent Medicine Clinic that houses the gender clinic in that our sample was more gender diverse. Most parents/caregivers identified as female (96.6%) and white or Caucasian (65.5%), with mean age of 47. Among parent/caregiver participants, 41.3% had a bachelor's degree or higher, 53.1% had private insurance and income was diverse. Tables 3 and 4 describe participant characteristics and care experiences/satisfaction with SCGC. Almost all participants found the Care Navigator to be “very” or “extremely” helpful (parents/caregivers 100%; youth 96%). Seventy percent of youth reported taking cross-sex hormones. The majority of participants felt “very” or “extremely” happy with hormone therapy (parents 77%; youth 90%). Almost all participants reported being “very” or “extremely” happy with overall care experiences at SCGC (parents/caregivers 96%; youth 94%), feeling that SCGC made it easy to access care (parents/caregivers 97%; youth 94%), and feeling supported by staff (parents/caregivers 96%; youth 100%). Most understood the next steps in care (parents/caregivers 90%; youth 94%) and felt the amount of information received was “just right” (parents/caregivers 93%; youth 100%).

**Table 3.** Demographics of Participants

Characteristic	Parents (*n*=29)	Youth (*n*=33)
	Mean (SD) or *N* (%)	Mean (SD) or *N* (%)
Age	46.61 (7.02)	15.78 (2.45)
Gender identity		
Male	1 (3.45)	
Female	28 (96.55)	
Transgender male		20 (55.56)
Transgender female		14 (38.89)
Nonbinary/gender fluid		2 (5.56)
Sex assigned at birth		
Male		14 (40)
Female		21 (60)
Marital status		
Single (never married)	3 (10.34)	
Married	20 (68.97)	
Divorced	5 (17.24)	
Separated	1 (3.45)	
Race		
White or Caucasian	19 (65.52)	18 (51.43)
Latinx	2 (6.9)	4 (11.43)
Other	3 (10.34)	3 (8.57)
More than one race	5 (17.24)	10 (28.57)
Annual household income		
<$50,000	9 (33.33)	
$50,000–$100,000	8 (29.63)	
>$100,000	10 (37.04)	
Parental education level		
High school or less	2 (6.9)	
Some college	15 (51.72)	
Bachelor's or higher	12 (41.38)	
Insurance type		
Private		17 (53.13)
Medicaid or other public		12 (37.5)
No insurance		2 (6.25)
Both private and public		1 (3.13)

SD, standard deviation.

**Table 4.** Care Experiences and Satisfaction

Variable	Parents (*n*=29)^a^	Youth (*n*=33)^a^
	Mean (SD) or *N* (%)	Mean (SD) or *N* (%)
How found clinic		
Doctor	13 (44.83)	10 (31.25)
Therapist	4 (13.79)	10 (31.25)
Friend or community member	1 (3.45)	3 (9.38)
Child or parent	1 (3.45)	9 (28.13)
Media	8 (27.59)	5 (15.63)
Online	3 (10.34)	6 (18.75)
Other	2 (6.9)	0 (0)
Child talked to Care Navigator		
Yes	25 (96.15)	27 (90)
No	1 (3.85)	3 (10)
Helpfulness of Care Navigator (1–5)	4.6 (0.5)	4.44 (0.7)
Very helpful (4)	10 (40)	12 (44.44)
Extremely helpful (5)	15 (60)	14 (51.85)
Child had readiness assessment^b^		
Yes	22 (84.62)	18 (85.71)
No	4 (15.38)	3 (14.29)
Happiness with assessment (1–5)^b^	4.14 (0.65)	3.88 (1.05)
Very happy (4)	12 (57.14)	7 (41.18)
Extremely happy (5)	6 (28.5)	5 (29.41)
Child receiving ongoing mental health therapy^b^		
Yes	21 (72.41)	23 (79.31)
No	8 (27.59)	6 (20.69)
Happiness with ongoing therapy (1–5)^b^	4.05 (0.78)	4.17 (0.78)
Very happy (4)	8 (42.11)	12 (52.17)
Extremely happy (5)	6 (31.58)	8 (34.78)
Child has taken puberty blockers^b^		
Yes	3 (11.11)	1 (3.7)
No	24 (88.89)	26 (96.3)
Child has taken cross-sex hormones^b^		
Yes	15 (53.57)	20 (71.43)
No	13 (46.43)	8 (28.57)
Happiness with cross-sex hormones (1–5)^b^	4 (0.71)	4.55 (0.69)
Very happy (4)	7 (53.85)	5 (25)
Extremely happy (5)	3 (23.08)	13 (65)
Child received gender-affirming surgery^b^		
Yes	0 (0)	0 (0)
No	28 (100)	29 (100)
Overall happiness with SCGC (1–5)	4.66 (0.55)	4.59 (0.71)
Very happy (4)	8 (27.59)	8 (25)
Extremely happy (5)	20 (68.97)	22 (68.75)
SCGC makes it easier to get care (1–5)	4.76 (0.79)	4.67 (0.6)
Agree (4)	3 (10.34)	7 (21.21)
Strongly agree (5)	25 (86.21)	24 (72.73)
Felt supported (1–5)	4.72 (0.8)	4.85 (0.36)
Agree (4)	4 (13.79)	5 (15.15)
Strongly agree (5)	24 (82.76)	28 (84.85)
Understood next steps (1–5)	4.45 (0.91)	4.5 (0.72)
Agree (4)	8 (27.59)	11 (34.38)
Strongly agree (5)	18 (62.07)	19 (59.38)
Amount of information		
Too little	1 (3.45)	0 (0)
Just right	27 (93.1)	33 (100)
Too much	1 (3.45)	0 (0)

^a^ Total number of responses do not add up to *n*=33 or *n*=29 because “I don't know” and “Skip” were options for all questions and there were older youth (age 18+) who did not have a parent enrolled in this study.

^b^ Only children 13 and older (*n*=29) were asked about the services they had received.

Qualitatively, we identified three main themes. We briefly describe each theme as follows along with representative quotes and participants' pseudonyms.

### Affirming clinical environment

Overall, participants described their experiences at SGCC as overwhelmingly positive. They frequently described feeling welcomed and affirmed. Participants reported appreciating respectful care delivery and the consistent use of patients' preferred name and pronouns.

I'm just feeling myself getting emotional. To have my child have a place he feels safe, and secure, and non-judged, and understanding the compassion that we feel when we're there, that's priceless to me, as a parent. I truly am grateful for your clinic. There's not enough of them out there. When I look in your waiting room and see other kids liked mine, I just think I'm so glad that they have you. Really. Truly.

- Sheryl, White, biological parent of 13-year-old transmasculine youth

One youth participant described their experience at the SCGC and specifically appreciated efforts to create a safe space:

I liked that it was a safe space to express myself and not feel judged.

- Cory, 17-year-old, White transmasculine youth

### Access and scheduling barriers

Despite overall happiness with SCGC, there were barriers. Multiple participants expressed frustration with the mental health assessment process and felt it negatively impacted receipt of timely gender-affirming medical. Participants specifically described the lack of accessible mental health providers outside of SCGC. Some participants had difficulty scheduling mental health assessment appointments, whereas some found the geographic distance to be a barrier. Another barrier was the length of time between the readiness assessment and hormone initiation.

But the only thing that has been a bit of a source of frustration only is that we came there wanting hormones, and I know we weren't going to get them the first visit, but not really understanding that it was going to take months of getting these appointments done before we could get there. And it's been frustrating because they're so booked up and we can't get through the process for that piece sooner.

- Drew, White 20-year-old transmasculine youth

One parent/caregiver describes her experience attempting to support her child in accessing hormone replacement therapy and specifically barriers related to the mental health assessment process.

She's been going through this journey for a while now, and there's just all these delays. And then delaying getting into the mental health visits because they're so busy, and you have to have all these things before you can start anything, and I think it's just continued setbacks and frustrations because she desperately wanted to start hormones a long time ago. And I didn't know anyone to take her to.

- Trish, White, biological parent of a 16-year-old transfeminine youth

### Experiences with Care Navigator

The presence of a Care Navigator was integral to youth/parents/caregivers feeling supported during a process fraught with barriers and emotional stress. Multiple interviewees described the resources provided by the Care Navigator as very useful.

[The Care Navigator]'s communication skills, resources, words of wisdom, and compassion has been outstanding. I've never had a phone conversation with anyone in the medical field and felt so supported. I cannot say enough good things about her.

- Jenna, White, biological parent of a 15-year-old transmasculine youth

She sent us emails and gave us papers that had links to different courthouses and documentations that we would need to get completed, in order to change the name and gender marker on official documents. And that was really helpful. I had been on a couple other different websites, and I was looking at it and it seemed daunting, so it was helpful that she had it there. And it was laid out in a way that was easy to follow and understand.

- Elaine, White parent of a 17-year-old transfeminine youth

## Discussion

Patient/family satisfaction is an integral component of delivering patient-centered care.^7^ The few studies that have examined patient satisfaction related to gender care were conducted with adult participants only.^8,9^ Results indicate an overwhelmingly positive response to the affirming environment of SCGC, a phenomenon also noted in a recent study of satisfaction with an adult gender dysphoria clinic.^8^ Satisfaction with our Care Navigator echoes sentiments expressed by patients in other settings.^10^

Mental health assessments and their impact on timely medical care arose as a theme; we received the lowest satisfaction scores in this area. Guidelines recommend a mental health consultation to assess for coexisting mental health conditions before pubertal blockers and cross-sex hormone therapy for TGNC youth.^11^ Mental health assessments are important to provide comprehensive care to TGNC youth and address coexisting mental health conditions that could impact gender-affirming treatment. To our knowledge, very little has been published exploring the mental health assessment process for TGNC youth engaged in gender care and specifically focused on refining the process to account for the spectrum of mental health needs of some TGNC youth. More research is needed to better understand the scope of who needs an assessment and to inform the development of more specific guidelines on completing the assessment process. These efforts may be incredibly beneficial to mental health providers (and medical providers) who may be less familiar with working with transgender youth. In our clinic, we find that there are fewer initial barriers in moving forward with gender-affirming medical care when a patient's own therapist can complete an easy online questionnaire for that young person's readiness assessment. If patients do not have a therapist, our SCGC Care Navigator helps patients find a community therapist through their insurance or helps them get scheduled with our clinic's mental health provider for an assessment (Table 5). Furthermore, given the shortage of mental health providers trained to work with TGNC youth, more mental health providers need to be trained in the community and incorporated into gender clinics.

**Table 5.** Mental Health Assessment Questionnaire

Therapist questions
What is the patient's preferred name and pronouns?
What is the patient's legal name, sex at birth, and gender identity?
Has the patient ever sought/received mental health therapy in the past? If so, when and how long were they in therapy?
Please describe the patient's gender journey.
When did they begin to explore their gender?
What challenges and supports have they encountered?
What are their hopes for affirming their gender in the future?
Who actively supports the patient in their gender transition?
For example, are there specific family members, friends, school, faith community, community groups, and professional helpers?
What cultural considerations may be helpful to be aware of when working with this patient, including factors that may be supportive or challenging to gender transition?
For example, religion, race, ethnicity, and other dimensions of diversity.
What are the strengths of this patient that you would like to highlight?
What are some potential barriers to care?
For example, lack of parental consent, transportation to appointment, insurance coverage, and uncertainty about desired interventions.
What coping skills and resources has the patient developed to address potential barriers?
Please describe the patient's mental health history, including how gender dysphoria may have impacted mental health.
For example, treatment history, recent changes, coping skills and supports, and plans to support safety.
Does the patient meet the criteria for a diagnosis of gender dysphoria?
Do you believe this patient could benefit from evaluation or resources around autism spectrum disorder?
If so, please elaborate.
What benefits do you believe the patient would experience as a result of pursuing gender-affirming medical care?
What, if any, concerns do you have about this patient pursuing gender-affirming medical care?
What recommendations would you like to share for resources and supports as this patient seeks gender-affirming medical care?
For example, ongoing individual therapy, consult for mental health medication, dialectical behavioral therapy group, and peer support group.

Study limitations include small sample size, study location at a single site, age range of youth participants (most participants were not candidates for puberty blockers), and a majority white sample, all of which limit generalizability. More research is urgently needed to understand engagement in and satisfaction with aspects of gender-affirming care among TGNC youth of color and their parents/caregivers. Nevertheless, this study sheds light on changes that all clinics could implement. A nonaffirming environment is cited frequently as a barrier to care for TGNC youth and adults.^1,8^ In response, we are actively trying to increase access to the SCGC, namely by hiring additional providers. We are also conducting trainings and other educational opportunities for outside medical and mental health providers interested in providing gender-affirming care. We have formed a group of community experts who are tasked with incorporating patient feedback from this study and formulizing the readiness assessment process for all patients seeking gender-affirming care.

This study reveals high satisfaction with our model of gender-affirming care, which is multidisciplinary coordinated care for transgender youth and their families, and addresses other barriers to care. As access remains a significant barrier, more coordinated care programs are needed and should include outreach and training in gender-affirming medical and mental health care for community providers.

## Abbreviations Used

SCGC: Seattle Children's Gender Clinic
TGNC: transgender/gender nonconforming
